# Human Milk sIgA Molecules Contain Various Combinations of Different Antigen-Binding Sites Resulting in a Multiple Binding Specificity of Antibodies and Enzymatic Activities of Abzymes

**DOI:** 10.1371/journal.pone.0048756

**Published:** 2012-11-02

**Authors:** Sergey E. Sedykh, Valentina N. Buneva, Georgy A. Nevinsky

**Affiliations:** Institute of Chemical Biology and Fundamental Medicine, Siberian Division of Russian Academy of Sciences, Novosibirsk, Russia; University of South Florida College of Medicine, United States of America

## Abstract

In the classic paradigm, immunoglobulins are monospecific molecules that have stable structures and two or more identical antigen-binding sites. However, we show here for the first time that the sIgA pool of human milk contains, depending on the donor, only 35±5% λ-sIgAs, 48±7% κ-sIgAs, and 17±4% of chimeric λ-κ-sIgAs. sIgA preparations contained no traces of canonical enzymes. However, all sIgA fractions eluted from several specific affinity sorbents under the conditions destroying even strong immune complexes demonstrated high catalytic activities in hydrolysis of ATP, DNA, and oligosaccharides, and phosphorylation of proteins, lipids, and oligosaccharides. Sequential re-chromatographies of the sIgA fractions with high affinity to one affinity sorbents on the second, third and then fourth affinity sorbents bearing other immobilized antigens led to the distribution of Abs and all catalytic activities all over the profiles of these chromatographies; in all cases some fractions eluted from affinity sorbents only under the conditions destroying strong immune complexes. *In vitro*, only an addition of reduced glutathione and milk plasma containing no Abs to two sIgA fractions with different affinity for DNA-cellulose led to a transition of up to 11–20% of Ab from one fraction to the other. Our data are indicative of the possibility of half-molecule exchange between different IgA and sIgA molecules. In addition, it cannot be excluded that during the penetration of IgAs through the specific milk barrier, the secretory component (S) and the join chain (J) can combine molecules of dimeric H_2_L_2_ λ-IgAs and κ-IgAs against different antigens forming many different variants of H_4_L_4_SJ sIgA molecules. Therefore, some chimeric molecules of sIgA can contain from two to four HL-fragments to various antigens interacting with high affinity with different sorbents and catalyzing various chemical reactions. Our data essentially expand the ideas concerning explanation of the phenomenon of polyspecificity and cross-reactivity of Abs.

## Introduction

Analysis of published data suggests that pregnant women may be directly immunized through a specific response of their immune system to certain compounds of viral, bacterial or food origin that can efficiently stimulate production of different antibodies (Abs). Immunization of animals by direct injection of antigens (mainly proteins) into the bloodstream or by oral administration no more than 1–3 months before delivery leads to the production of anti-antigen Abs, which then may be detected in the milk at high concentrations [1]. Human milk contains various types of Abs (IgG, IgM, IgA and sIgA), of which sIgA is the major component (> 85–90%) [2], [3].

During pregnancy and immediately after delivery, women are very often characterized by immune processes similar to those in autoimmune patients ([4]–[6] and references therein). Many autoimmune pathologies can be “activated” or “triggered” in clinically healthy women during pregnancy and soon after childbirth [7], [8].

During the last two decades it has become clear that auto-antibodies (auto-Abs) from the sera of patients with different autoimmune and several viral diseases can possess enzymatic activities (reviewed in [4]–[6], [9]–[11]). Similarly to artificial abzymes against analogs of transition states of catalytic reactions [4], naturally occurring Abzs may be Abs raised directly against the enzyme’s substrates acting as haptens and mimicking transition states of catalytic reactions [4]–[6], [9]–[11]. On the other hand, antiidiotypic Abs can be induced in autoimmune diseases by a primary antigen and may show some of its features including the catalytic activity [12], [13].

Natural Abzs hydrolyzing DNA, RNA, polysaccharides, oligopeptides, and proteins are described from the sera of patients with several autoimmune and viral diseases (for review see [4]–[6]). In addition, convincing evidence was provided using different approaches including several strict criteria that DNase, RNase [14]–[16], amylase [17], ATPase [18], and protease [19] as well as protein kinase [20], lipid kinase [21], and polysaccharide kinase [22], [23] enzymatic activities are intrinsic to human milk IgGs and sIgAs. In contrast to canonical enzymes, milk IgG and sIgA abzymes possess a unique capability to phosphorylate milk proteins and tightly bound with these Abs minor lipids and oligosaccharides having unusual structure in the presence of [^32^P]*ortho*phosphate [20]–[23].

Interestingly, the relative blood abzyme activities significantly increase after delivery and at the beginning of lactation [18], [24]. Nevertheless, enzymatic activities of Abs from the milk of lactating women are 5–600-fold higher than those from the sera of the same women [18], [24]. In addition, the DNase activity of Abzs from blood of healthy pregnant women was 4–5-fold lower than that from pregnant women with pronounced autoimmune thyroiditis [24].

There was a common belief that IgGs and IgAs are monospecific molecules having stable structures and two identical antigen-binding sites [25]–[28]. Recently, it was shown that human IgG4 antibodies are dynamic molecules that exchange Fab arms by swapping a heavy chain and the attached light chain (half-molecule) with a heavy–light chain pair from another molecule, which results in bispecific Abs [25]–[28]. Reduced glutathione (GSH) together with some blood proteins stimulates the exchange *in vitro* leading to formation of hybrid molecules from two different IgG4 [25]–[28]. The formation of bispecific IgG4 was also revealed *in vivo* [25].

It was recently shown, that in human milk IgGs to different antigens as well as kappa-and lambda-IgGs undergo extensive half-molecule exchange [29]. Chimeric kappa-lambda-IgGs consisted of ∼74% IgG1, ∼16% IgG2, ∼5% IgG3 and ∼5% IgG4.

Interestingly, there was no data concerning possibility of the IgG4 exchange by only light or heavy chains. Therefore, we have obtained FITC-labeled preparations of separated light and heavy chains of milk IgGs [29]. Then a possibility of non-modified IgG preparation labeling after its incubation with isolated FITC-L- and FITC-H-chain preparations in the absence and in the presence of plasma and GSH was analyzed. It was shown, that in contrast to the exchange of intact IgGs, incubation of IgGs with separated FITC-modified light as well as heavy chains does not lead to the exchange; there was not revealed FITC-labeled intact IgGs by SDS-PAGE [29].

It is known, that antigen-binding site of Abs is usually formed by variable parts of both light and heavy chains. In abzymes, the catalytic center and a part of the binding site are usually located on the light chain, while the heavy chain is more often responsible for the specific antigen recognition and increased antigen affinity for Abs [4]–[6]. It is likely that exchanging only light or only heavy chains is suppressed (or prohibited), since it can lead to the formation of Abs with abnormal non-functional combinations of variable parts of H- and L-chains corresponding to different antigens. According to [25]–[28], disulphide isomerase and/or FcRn protein in the case of IgG4 can stimulate the exchange. It cannot be excluded that specific enzymes (or non-catalytic proteins) can recognize exclusively HL-fragments (but not individual L- or H-chains) of Abs and therefore can stimulate only the half-molecule exchange.

As the result of the exchange, all IgG fractions eluted from several specific affinity sorbents under the conditions destroying strong immune complexes demonstrated high catalytic activities in hydrolysis of ATP, DNA, oligosaccharides, phosphorylation of proteins, lipids, and oligosaccharides. *In vitro*, the incubation of IgGs in reaction mixture containing only reduced glutathione (GSH) or only milk plasma did not lead to the half-molecule exchange [29]. At the same time, after the addition of both GSH and milk plasma to the exchange mixtures containing two IgG fractions with different affinity for DNA-cellulose, a transition of 25–60% of Ab of one fraction to the other fraction was observed.

These data indicate for half-molecule exchange between milk IgGs of various subclasses, raised against different antigens (including abzymes), which explains the observed catalytic polyspecificity and cross-reactivity of these milk IgGs [29].

Human milk contains IgG, IgM, IgA, and sIgA, of which sIgA is the major component (> 85–90%) [30]. It is known that the molecular masses, structure and sources of human milk IgGs, IgAs, and sIgAs are different [1]–[3]. IgA of patients with autoimmune and viral diseases and sIgA of human milk are usually significantly more catalytically active than IgG abzymes [4]–[6]. Thus, there may be a significant difference in IgGs, IgAs, and sIgAs of human milk. Therefore, it was very interesting to compare structural peculiarities, possible cross-reactivity and difference in an efficiency of a possible exchange leading to the formation of Abs not only with binding, but also with catalytic polyspecificity in the case of human milk IgG and sIgA antibodies. Taking into account the absence of IgG exchanging only light or only heavy chains, it was reasonable to suggest that sIgAs also may exchange only half-molecules.

In this report we have used several methods to provide the first evidence that molecules of human milk sIgAs can undergo specific extensive exchange and consist of HL-fragments with various antigen-binding sites resulting in a multiple binding and enzymatic activities of molecules of Abs and abzymes, respectively.

## Results

**Figure 1:**
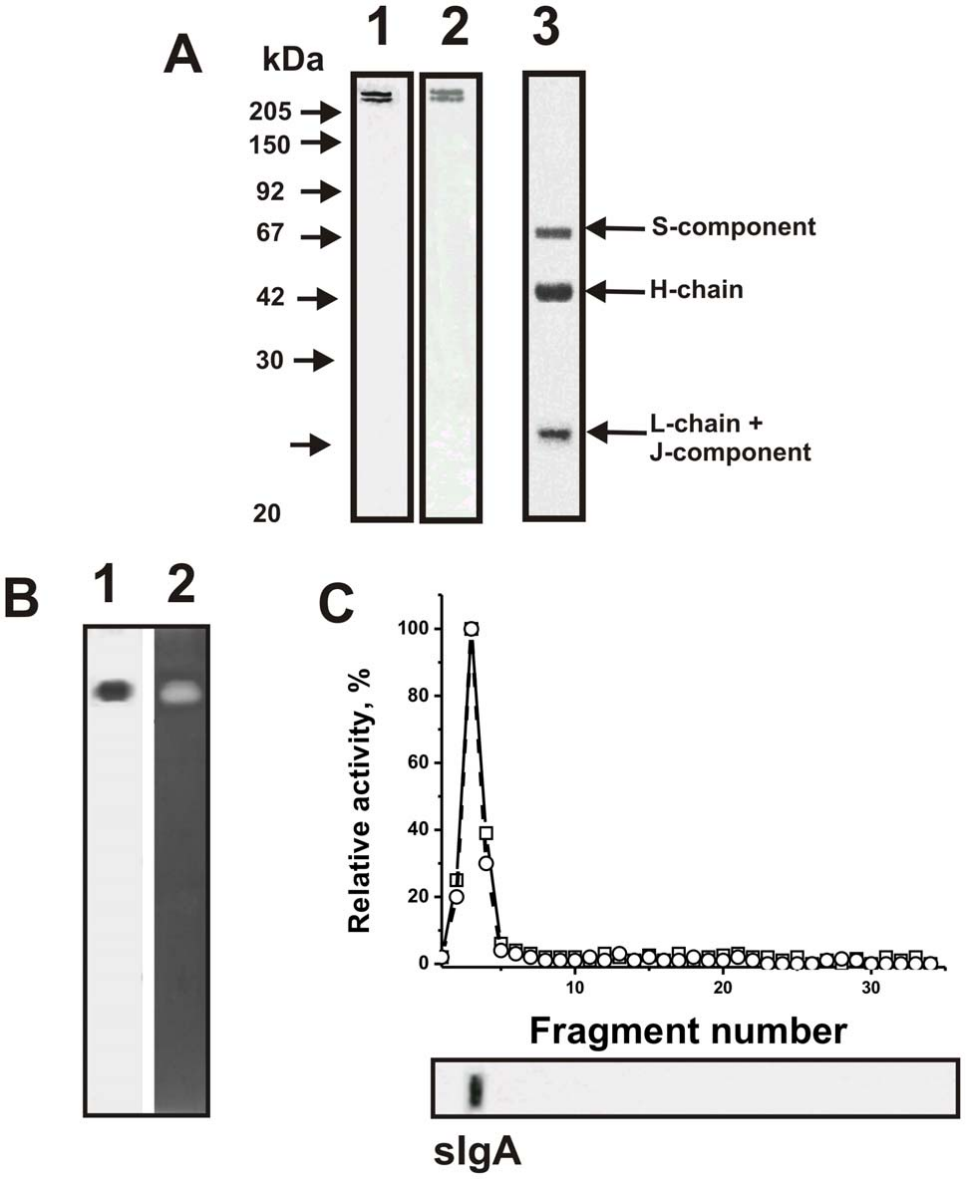
Analysis of the homogeneity of sIgA_mix_ preparation (5 µg) from milk of mothers by SDS-PAGE in a nonreducing 4–16% gradient gel (lane 1) or a reducing 12.5% gel (lane 3) followed by silver staining (A). The arrows indicate the positions of molecular mass markers. Immunoblotting analysis of proteins (lane 2; corresponds to silver staining, lane 1) interacting with mouse polyclonal IgGs against human sIgAs (A). In-gel assay of DNase activity of sIgA_mix_ preparation under nonreducing conditions (B); 10 µg sIgA_mix_ was used (lanes 1 and 2). DNase activity was revealed by ethidium bromide staining as a dark band on the fluorescent background (lane 2). A part of the gel was stained with Coomassie R250 to show the position of intact sIgA_mix_ (lane 1). SDS-PAGE analysis of ATPase and amylase activities of intact sIgA_mix_ (C). Before electrophoresis, sIgA_mix_ (10 µg per sample) was incubated under nonreducing conditions. After electrophoresis, the gels were incubated under special conditions for protein refolding (see Materials and methods). Then, the relative ATPase (□) and amylase (○) activities were analyzed using extracts of 2-3-mm fragments of a longitudinal gel slice (C). Maximal activity of one fraction in the hydrolysis of ATP (or oligosaccharide) was taken for 100%. In each experiment the second longitudinal slice of the same gel was silver-stained; the arrows indicate the positions of intact sIgA (D). For details, see Materials and methods.

In this work, homogeneous polyclonal sIgAs were purified from human milk using conditions destroying nonspecific interactions and strong immune complexes as in [19]–[23]. In agreement with previously published data [16]–[18], [20], elution of proteins from Protein A-Sepharose with acidic buffer, pH 2.6, produces an electrophoretically homogeneous mixture of oligomeric ∼370 kDa sIgAs (1 S-, 72 kDa; 4 heavy-, 62 kDa; 4 light-, 23 kDa and 1 J-chain, 23–26 kDa) and ∼300 kDa sIgA2 in which light chains are not linked covalently to the oligomer by disulfide bonds and they loose light subunit under drastic conditions of Ab purification (Fig. 1A; lane 1); both sIgA and sIgA2 positively reacted with anti-IgA Abs during immunoblotting (Fig. 1A, lane 2). Only three bands were seen in reducing gel where the L-chain and J-component comigrate (Fig. 1A, lane 3). As it was shown previously [20], all four components of the 370 kDa form of sIgA can be identified using 2-dimensional electrophoresis (two coordinates: molecular size and isoelectric point).

For some experiments we have used a mixture of equal amounts of sIgAs from the milk of five donors (sIgA_mix_). To prove that the DNase and other analyzed activities of sIgAs used in this study belongs to the Abs and is not due to co-purifying enzymes, we have applied several previously developed strict criteria [4]–[6], [10]. They may be summarized as follows: a) according to SDS-PAGE the sIgA_mix_ preparation contained only sIgA1 and sIgA2 (Fig. 1); b) gel filtration of sIgA_mix_ under conditions dissociating strong noncovalent complexes in an acidic buffer (pH 2.6) did not eliminate the activities analyzed, and the peaks of all activities tracked exactly with the intact sIgA_mix_; c) immobilized mouse polyclonal IgGs against the human sIgAs completely absorbed all activities, and these activities corresponded only to the peak of sIgAs eluted with an acidic buffer (data not shown).

In addition, to exclude possible artefacts due to hypothetical traces of contaminating DNases, sIgA_mix_ was subjected to SDS-PAGE in a gel co-polymerized with calf thymus DNA, and its DNase activity was detected by incubating the gel in the standard reaction buffer. Ethidium bromide staining of the gels after the electrophoresis and refolding of sIgAs revealed sharp dark band against a fluorescent background of DNA only in the position of intact sIgA_mix_ (Fig. 1B, lane 2).

To exclude possible artefacts due to hypothetical traces of contaminating ATPases and amylases, sIgA_mix_ used in this study was separated by SDS-PAGE and its activities were detected after extraction of proteins from the separated gel slices (Fig. 1C). Since SDS dissociates all protein complexes, the detection of DNase (Fig. 1B), ATPase, and amylase (Fig. 1C) activities in the gel region corresponding only to intact sIgA, together with the absence of any other bands of the activity or protein, provides direct evidence that the used intact sIgAs preparations posses all these activities and are not contaminated by canonical enzymes.

It was shown previously that only milk IgGs and sIgAs (but not any canonical enzymes) can use *ortho*phosphate as a very unique substrate in the phosphorylation of proteins, lipids and oligosaccharides [20]–[23]. Therefore, we have used in this study [^32^P]*ortho*phosphate as a very specific donor of phosphate group acting only in the case of milk abzymes; sIgAs used in this study possessed protein, lipid an oligosaccharide kinase activities. Thus, similarly to previously published findings [16]–[17], [19]–[22], it was shown that the sIgA preparations used in this study contain subfractions efficiently hydrolyzing DNA, ATP and oligosaccharides; they were able to phosphorylate proteins, as well as oligosaccharides and lipids that were tightly bound to these Abs.

**Figure 2:**
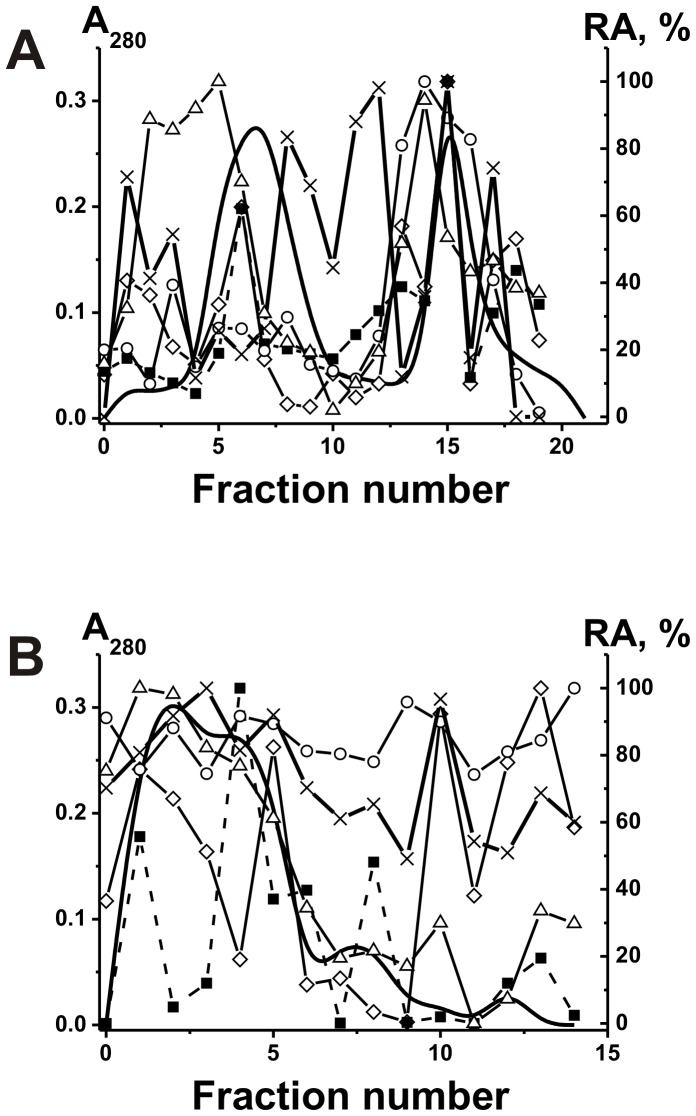
Affinity chromatography of milk sIgA-1 preparation from first donor on DNA-cellulose and ATP-Sepharose. sIgA-1 preparation was chromatographed on DNA-cellulose (A) and ATP-Sepharose (B) in standard conditions: (–), absorbance at 280 nm; symbols correspond to the relative catalytic activities (RA) in the hydrolysis of DNA (▵), ATP (○), oligosaccharides (×) as well as phosphorylation of lipids (▪) and polysaccharides (⋄) tightly bound with sIgAs. Depending on the RA and reaction analyzed, the reaction mixtures were incubated for 0.5–2 h and then the RAs were normalized to the standard conditions and the RA of the fraction with the highest activity was taken for 100%. The average error in the initial rate determination from two experiments in each case did not exceed 7–10%. See Materials and methods for other details.

Polyclonal IgGs and sIgAs with different catalytic activities are usually very heterogeneous in their affinity for different specific substrates and can be separated into many subfractions by chromatography on specific affinity sorbents [4]–[6], [9], [14]–[16], [18]–[21]. We have obtained sIgAs from the milk of five women and first analyzed their affinity for DNA and ATP by chromatography on DNA-cellulose and ATP-Sepharose, respectively. When individual IgAs were eluted from DNA-cellulose and ATP-Sepharose with a NaCl concentration gradient (0–3 M) and 3 M MgCl_2_, the protein and DNase activity were distributed all over the profiles of these chromatographies. Fig. 2 demonstrates representative data for one (sIgA-1) of five individual sIgA preparations. In contrast to previous studies [6], [16], [18], [20], [21], we have analyzed relative catalytic activities (RAs) of the sIgA fractions eluted from DNA-cellulose not only in the hydrolysis of plasmid DNA, but also in the hydrolysis of ATP and oligosaccharides as well as in phosphorylation of proteins, lipids and oligosaccharides (Fig. 2A). Similar determination of the relative activities of Abs in different chemical reactions was performed after sIgA-1 preparation chromatography on ATP-, casein-, and phenyl-Sepharose; all these six activities were always distributed all over the chromatography profiles (for example, Fig. 2B).

**Figure 3:**
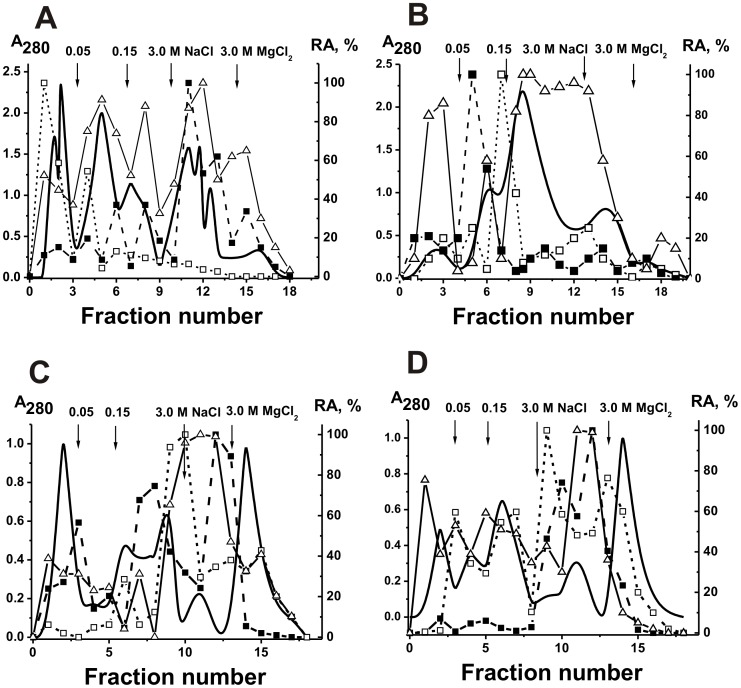
Sequential affinity chromatography of sIgA-2 and sIgA-3 preparations from second and third donors on different affinity sorbents. After chromatography of sIgA-2 and sIgA-3 preparations on DNA-cellulose (A and B, respectively), fractions 10–18 having high affinity to DNA-cellulose were combined and chromatographed on ATP-Sepharose (C and D, respectively): (–), absorbance at 280 nm; relative catalytic activities (RA) in the hydrolysis of DNA (▵), phosphorylation of casein (□) and lipids tightly bound with Abs (▪). Depending on the RA and reaction analyzed, the reaction mixtures were incubated for 0.5–2 h and then the RAs were normalized to the standard conditions and the RA of the fraction with the highest activity was taken for 100%. The average error in the initial rate determination from two experiments in each case did not exceed 7–10%. See Materials and methods for other details.

**Figure 4:**
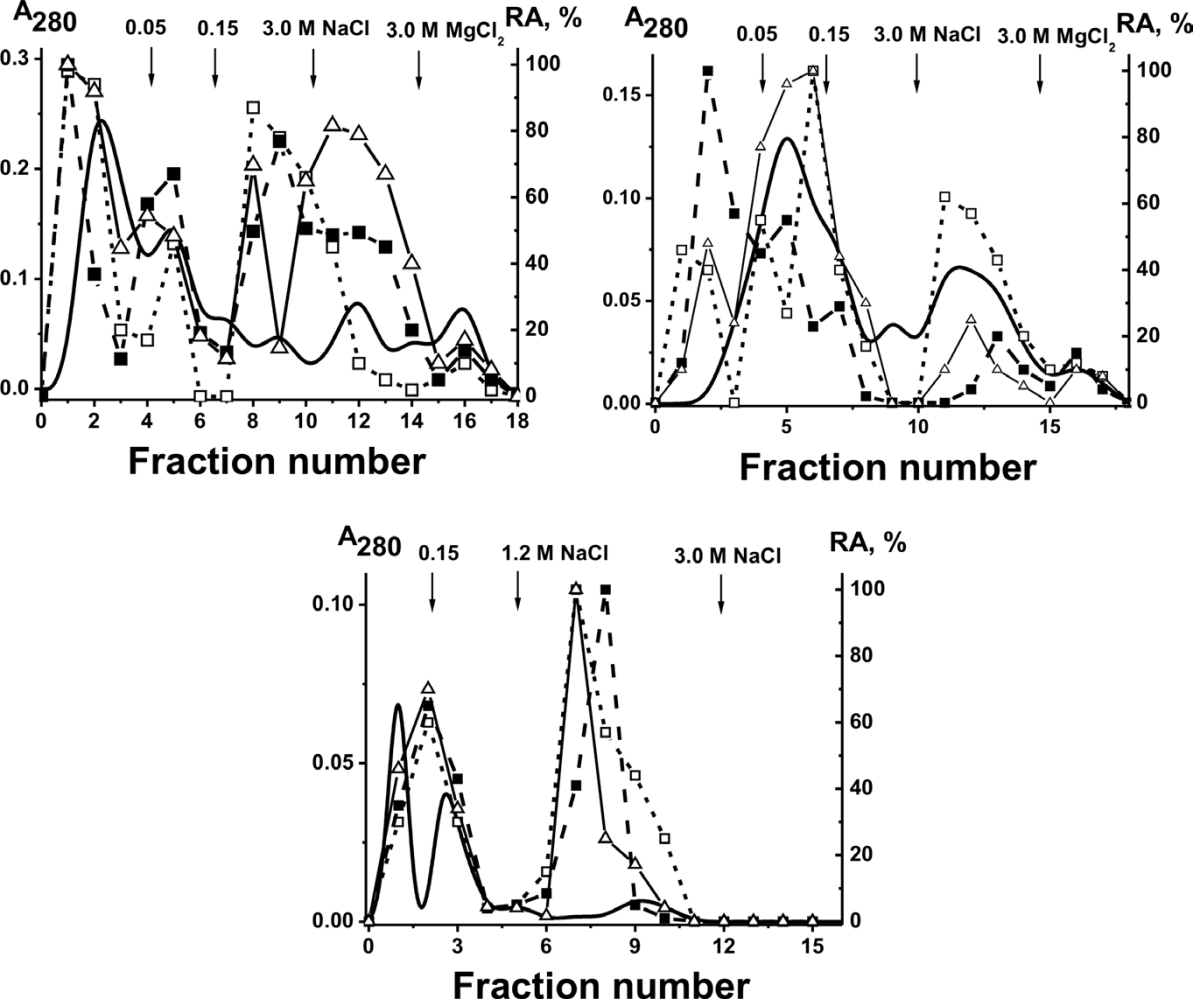
Sequential affinity chromatography of sIgA-2 and sIgA-3 preparations on casein-Sepharose and lipid-resin. After chromatography of sIgA-2 and sIgA-3 preparations on ATP-Sepharose (Figs 3C and 3D, respectively), fractions 10–18 having high affinity to ATP-Sepharose were combined and chromatographed on casein-Sepharose (Figs 4A and 4B, respectively). Panel C demonstrates chromatography on lipid-resin of a mixture of fractions 9–17 corresponding to sIgA-2 and sIgA-3 eluted from casein-Sepharose (Figs 3C and 3D): (–), absorbance at 280 nm; relative catalytic activities (RA) in the hydrolysis of DNA (▵), phosphorylation of casein (□) and lipids tightly bound with Abs (▪). Depending on the RA and reaction analyzed, the reaction mixtures were incubated for 0.5–2 h and then the RAs were normalized to the standard conditions and the RA of the fraction with the highest activity was taken for 100%. The average error in the initial rate determination from two experiments in every case did not exceed 7–10%. See Materials and methods for other details.

It was surprising, that all sIgA fractions including those eluted under the conditions of destroying of not only week, but also strong immune complexes of Abs with specific antigens (1–3 M NaCl and even 3 M MgCl_2_) demonstrated not only high DNase activity but also efficiently hydrolyzed ATP and oligosaccharide as well as phosphorylate casein, and tightly bound with Ab oligosaccharides and lipids (Figs 2A and 2B). Therefore, we tried to analyze affinity of individual sIgA-2 and sIgA-3 preparations having high affinity to DNA-cellulose (fractions 10–18; Figs 3A and 3B) to ATP-Sepharose (Figs 3C and 3D). In spite of some difference, the Ab optical density and all catalytic activities of these combined fractions of sIgA-2 and sIgA-3 preparations were revealed in all eluted fractions (Figs 3C and 3D). Then sIgA-2 and sIgA-3 fractions possessing high affinity to ATP-Sepharose (fractions 10–18, Figs 3C and 3D) were re-chromatographed on casein-Sepharose (Figs 4A and 4B). Again, sIgA-2 and sIgA-3 fractions demonstrated extreme heterogeneity in their affinity to casein and all activities were distributed all over the profiles of these chromatographies. Finally we mixed fractions 9–17 of sIgA-2 and sIgA-3 eluted from casein-Sepharose (Figs 4A and 4B) and chromatographed this combined Ab preparation on lipid-resin (Fig. 4C). One can see that again all eluted fractions contain sIgA possessing several different catalytic activities.

**Figure 5:**
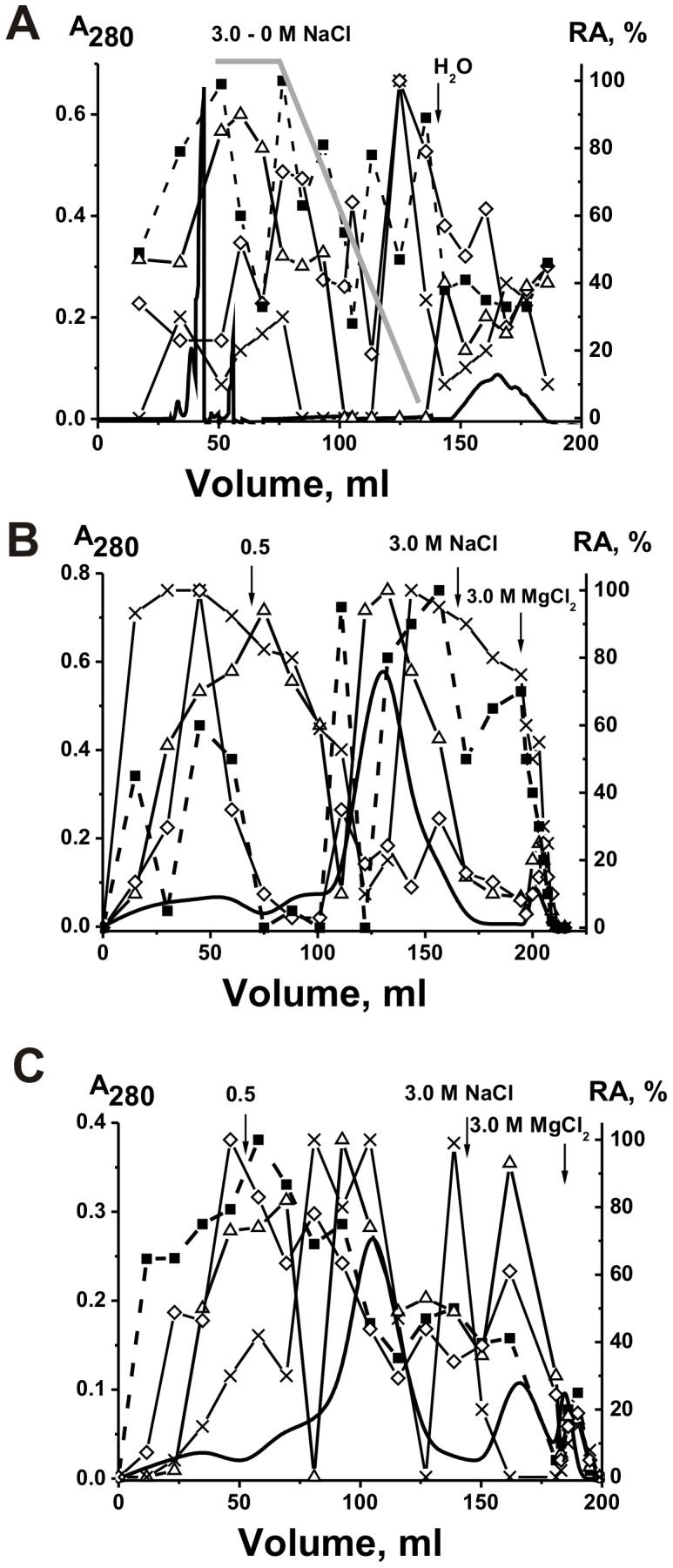
Sequential affinity chromatography of sIgA-2 and sIgA-3 preparations on phenyl-Sepharose and DNA-cellulose. Hydrophobic chromatography of sIgA_mix_ (equimolar mixture of Abs from five donors) on phenyl-Sepharose using reverse gradient of NaCl concentration (A). Chromatography of fractions 0–70 ml (B) and 160–190 ml (C) eluted from phenyl-Sepharose on DNA-cellulose: (–), absorbance at 280 nm; relative catalytic activities (RA) in the hydrolysis of DNA (Δ) and maltoheptaose (x) as well as phosphorylation of oligosaccharides (◊) and lipids (▪) tightly bound with Abs. Depending on the RA and reaction analyzed, the reaction mixtures were incubated for 0.5–2 h and then the RAs were normalized to the standard conditions and the RA of the fraction with the highest activity was taken for 100%. The average error in the initial rate determination from two experiments in each case did not exceed 7–10%. See Materials and methods for other details.

sIgA fractions containing tightly bound minor lipids and oligosaccharides in principle could posses higher affinity to hydrophobic sorbents. For further experiment we have used a mixture of equal amounts of sIgAs from the milk of five donors (sIgA_mix_). We tried to separate sIgA_mix_ fractions possessing different hydrophobicity by chromatography on Phenyl-Sepharose using reverse gradient of NaCl concentration (Fig. 5A). Less hydrophobic sIgA_mix_ fractions were eluted substantially under loading, while Abs with higher hydrophobicity by water, but sIgAs and their different catalytic activities were again distributed all over the profile of this chromatography. Similar result was observed after chromatography of sIgAs of the first and second peaks (fractions corresponding to fractions 0–70 and 160–190 ml, Fig. 5A) on DNA-cellulose (Figs 5B and 5C).

It is known that various canonical enzymes are usually eluted from the specific affinity sorbents as a single peak with the maximal activity corresponding to a fixed concentration of the eluting salt. The data indicated for extreme heterogeneity of milk polyclonal sIgAs in their affinity to various substrates and for a significant multiplicity of their catalytic activities.

Polyspecificity is defined as the ability of a given Ab molecule to bind a large panel of structurally diverse antigens. Some studies demonstrated the existence of a large number of monoclonal Abs that can bind to a variety of totally unrelated self and foreign antigens (for review see [31]). Therefore, it was proposed that the best explanation for the polyreactivity is that the antigen-binding ‘pocket’ of many Ab molecules may be flexible and can change conformation to accommodate different antigens [31]. From one side, one cannot exclude that that in the case of some Abs an increase in salt concentration can be associated with a change of Abs conformation leading to their binding polyspecificity. However, all nonspecific interactions between Abs (or canonical enzymes) and foreign ligands can usually be completely (or at least to a significant extent) destroyed by 0.2–0.5 M NaCl [4]–[6], [9], [31]–[34]. For example, we have previously shown that mouse monoclonal IgGs against ATP can interact with DNA but posses 3–4 orders of magnitude lower affinity to DNA then to Abs against DNA and they can be eluted from ATP-Sepharose by ≤0.05 M NaCl [5], [6], [33]. In addition, canonical enzymes can sometimes interact nonspecifically with foreign ligands demonstrating lower affinity then to specific substrates, but they usually cannot catalyze conversion of molecules of non-cognate compounds [32] (see below). Therefore, it was surprising that all sIgA fractions, including those eluted under the conditions destroying strong complexes of Abs with specific antigens (1–3 M NaCl and even 3 M MgCl_2_) before and after several sequential chromatographies on different affinity sorbents, not only demonstrated high DNase activity but also efficiently hydrolyzed ATP and oligosaccharides, and phosphorylated casein, oligosaccharides and lipids (Figs 2, 3, 4, 5). A known enzymes usually possess only one catalytic activity. All our attempts to separate abzymes with different individual catalytic activities were unsuccessful.

The 370 kDa milk sIgA consists of four heavy (H), four light (L), one secretory (S) and one join (J) chain (H_4_L_4_SJ) [30]. Therefore it was reasonable to suggest that human milk might contain not only monofunctional abzymes but also hybrid chimeric bifunctional sIgAs with different combination of HL fragments of H_4_L_4_SJ sIgA molecules, possessing affinity to different antigens and several catalytic activities.

Recently, the formation of bispecific IgG4 was revealed not only *in vitro*, but also *in vivo* [25]–[28]. Then, we have shown that human milk IgGs (IgG1-IgG4, λ-IgGs, and κ-IgGs) to different antigens undergo extensive half-molecule exchange [29].

**Figure 6:**
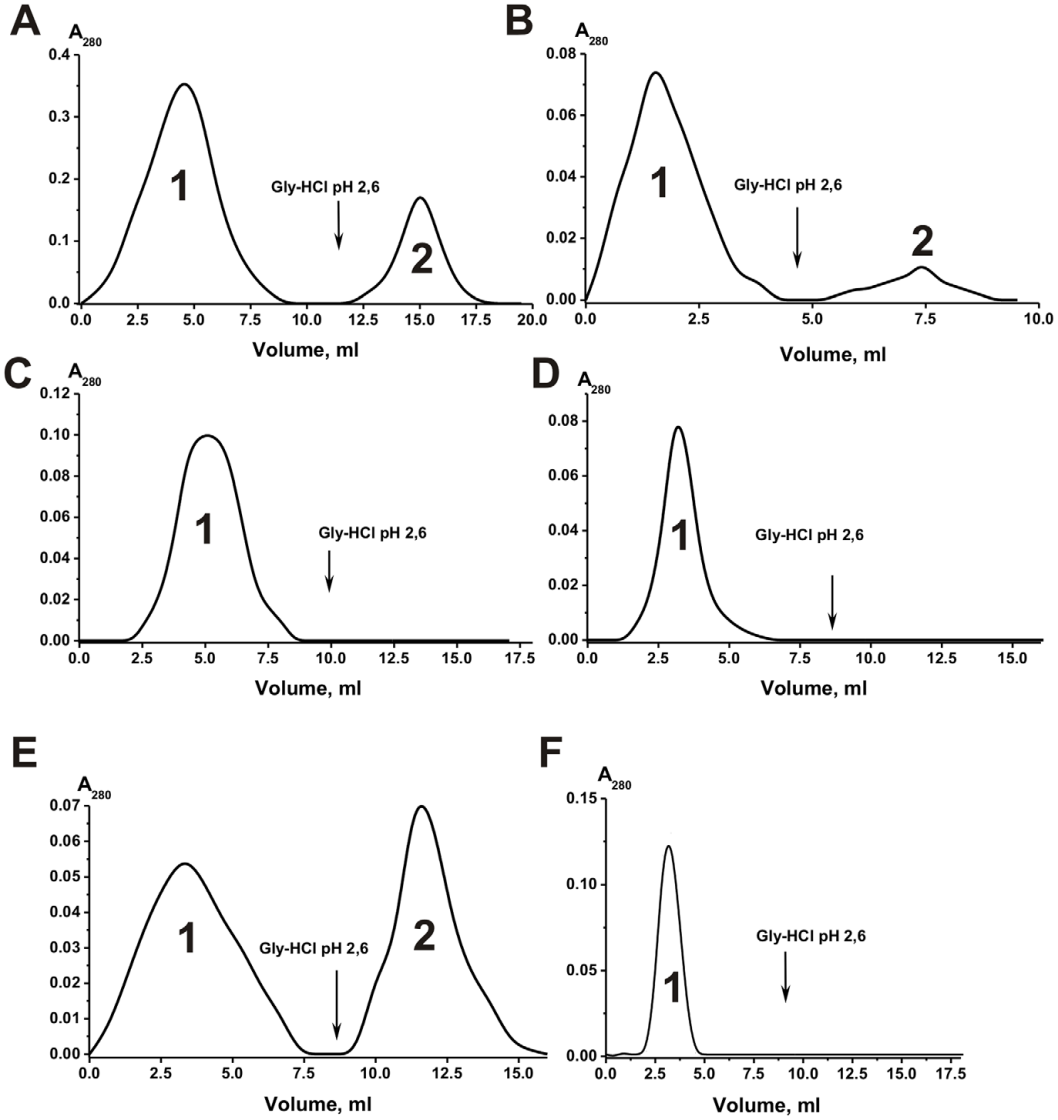
Affinity chromatography of milk sIgAs on different affinity sorbent. Affinity chromatography of milk sIgA-1 preparation on anti-κ-L-Sepharose under the conditions of over-saturation of the affinity capacity of the sorbent (A) and re-chromatography of the preparation eluted by acidic buffer (peak 2) on anti-λ-L-Sepharose (B) under the conditions of the excess of the affinity sorbent. Re-chromatography of κ-sIgA-1 and λ-sIgA-1 preparations containing no chimeric sIgAs on anti-λ-L-Sepharose (C) and anti-κ-L-Sepharose (D) respectively under the conditions of the excess of the affinity sorbents. After incubation of a mixture of equal amounts of purified λ- and κ-sIgAs for 24 h, λ+κ-sIgA_mix_ was subjected to standard affinity chromatography on Protein A-Sepharose and gel filtrated (data not shown). Then λ- and κ-sIgAs were separated by affinity chromatography of the mixture of λ- and κ-sIgAs (λ+κ-sIgA_mix_) on anti-κ-L-Sepharose (E). The preparation of κ-sIgAs purified on anti-κ-L-Sepharose was rechromatographed on anti-κ-L-Sepharose (E). In all cases: (–), absorbance at 280 nm (A_280_).

To reveal a possible existence milk sIgAs containing HL-fragments of different type, they were separated by affinity chromatography on Sepharose bearing immobilized monoclonal Abs to human Abs containing kappa- (anti-κ-L-Sepharose) and lambda-type (anti-λ-L-Sepharose) of light chains under the conditions of an excess of the affinity sorbent. To screen out nonspecific interactions, we then have isolated sIgA fractions on anti-κ-L-Sepharose and anti-λ-L-Sepharose under the conditions of over-saturation of the affinity capacity of the sorbents (for example, Fig. 6A). The sIgA fraction having affinity for anti-κ-L-Sepharose was re-chromatographed on anti-λ-L-Sepharose (Fig. 6B). Depending on the individual preparation, 26±4% of IgAs having affinity for anti-κ-L-Sepharose was bound by anti-λ-L-Sepharose. In addition, sIgA fraction having affinity for anti-λ-L-Sepharose was re-chromatographed on anti-κ-L-Sepharose; 67±5% of the Abs was eluted in the flow-through, while 33±5% were bound by immobilized anti-κ-Abs.

We have rechromatographed purified λ-sIgAs and κ-sIgAs contaning no admixture of chimeric λ-κ-sIgAs on anti-lambda- and anti-kappa-L-Sepharoses respectively (Figs 6C and 6D). It was shown that λ-sIgAs interacts only with anti-λ-L-Sepharose (Fig. 6C), κ-sIgAs only with anti-κ-L-Sepharose (Fig. 6D), while λ-κ-sIgAs with both of these affinity sorbents. Using ELISA, it was confirmed that λ-sIgAs and κ-sIgAs interact only with anti-lambda- and anti-kappa Abs respectively, while λ-κ-sIgAs with both of these antibodies.

Taken all these data together, it was calculated that depending on the milk donor pool of sIgA antibodies on average consists of 35±5% λ-sIgAs, 48±7% κ-sIgAs and 17.0±4% of chimeric λ-κ-sIgAs interacting with both anti-lambda- and anti-kappa-L-Sepharose.

The data indicated for an existence in the human milk of intact sIgAs containing only lambda- or kappa-light chains as well as chimeric λ-κ-sIgAs. However, one could not exclude that the exchange reaction can occur (at least to some extent) during sIgAs purification or some other manipulation with Ab preparations. Therefore, we have prepared a mixture of approximately equal amounts of purified λ- and k-sIgA preparations (λ+κ-sIgA_mix_ preparation containing no chimeric lambda-kappa-sIgAs) which was incubated for 24 h. Then, this mixture was subjected to standard affinity chromatography on Protein G-Sepharose, the flow-through fraction on Protein A-Sepharose followed by standard gel filtration under the conditions that remove non-specifically bound proteins similarly to purification of sIgAs from human milk. To reveal a possible chain exchange during purification procedures, the λ+κ-sIgA_mix_ preparation was separated for lambda- and kappa-sIgAs by affinity chromatography on Sepharose bearing immobilized monoclonal Abs to human Abs containing κ- and λ-type of light chains. For example, Fig. 6E shows the data for anti-λ-L-Sepharose. The preparations of λ-sIgAs purified on anti-λ-L-Sepharose were rechromatographed on anti-κ-L-Sepharose (for example, Fig. 6F), while κ-sIgAs on anti-λ-L-Sepharose. In contrast to sIgAs purified on anti-λ-L-Sepharose directly from the samples of milk total sIgAs, preparation of λ-sIgAs obtained using mixture of purified λ- and κ-sIgAs did not contained admixtures of κ-sIgAs (Fig. 6F). Similar result was observed for preparations of κ-sIgAs demonstrating no any admixtures of λ-sIgAs. In addition, using ELISA it was also shown that sIgAs purified on anti-λ-L-Sepharose do not contain κ-sIgAs, while sIgAs purified on anti-κ-L-Sepharose are free of λ-sIgAs. These data show that the exchange reactions do not occur during antibody purification and other standard manipulation with Abs. In addition, the findings demonstrate that specific anti-λ-L-Sepharose bind only λ-sIgAs while anti-κ-L-Sepharose only κ-sIgAs. At the same time, both affinity resins interact with chimeric sIgAs containing simultaneously lambda- and kappa-chains (Fig. 6B).

**Figure 7:**
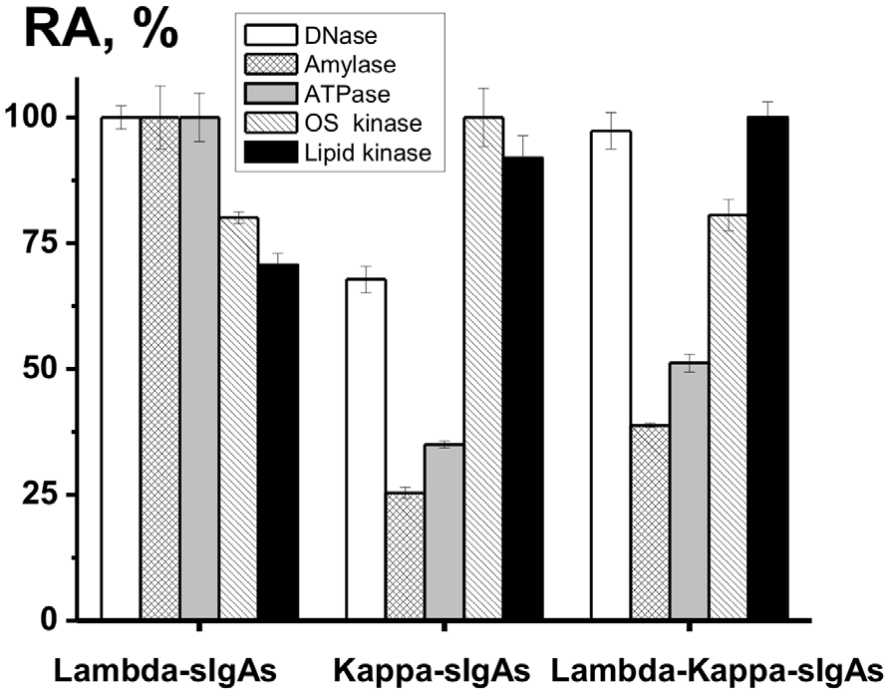
The relative activities of λ-sIgAs, κ-sIgAs containing no chimeric Abs, and λ-κ-sIgAs in the catalysis of different chemical reactions. The RA of the preparation with the highest activity in each reaction analyzed was taken for 100%. See Materials and methods for other details.

We have analyzed the relative activity of sIgAs of a different composition from one milk donor in the catalysis of several reactions (Fig. 7). Note, DNase, amylase, and ATPase activities of λ-sIgAs are higher than those for κ-sIgAs, while for lipid- and oligosaccharide kinase Ab activities a reverse situation is observed. The ratio of the RAs corresponding to the different catalytic activities was individual for λ-sIgAs, κ-sIgAs, and κ-λ-sIgAs. Interestingly, the relative activities for chimeric κ-λ-sIgAs in the case of several reaction analyzed were comparable with the average values for Abs containing only λ- or κ-light chains (Fig. 7).

Recently we have analyzed *in vitro* exchange by HL-fragments between molecules of intact and FITC-modified IgGs [29]. It was shown, that an addition of reduced glutathione together with milk plasma to two IgG fractions with different affinity for DNA-cellulose led to a transition of 25–60% of Ab of one fraction to the other fraction. Our data indicated for a half-molecule exchange between milk IgGs of various subclasses, raised against different antigens (including abzymes), which explains the catalytic polyspecificity and cross-reactivity of these IgGs. At the same time, since in contrast to IgGs, sIgA molecules contain secretory components (S) and join chain (J), it was possible to expect to some extent significant impediment of the exchange by HL-fragments between two different Ab molecules.

**Figure 8:**
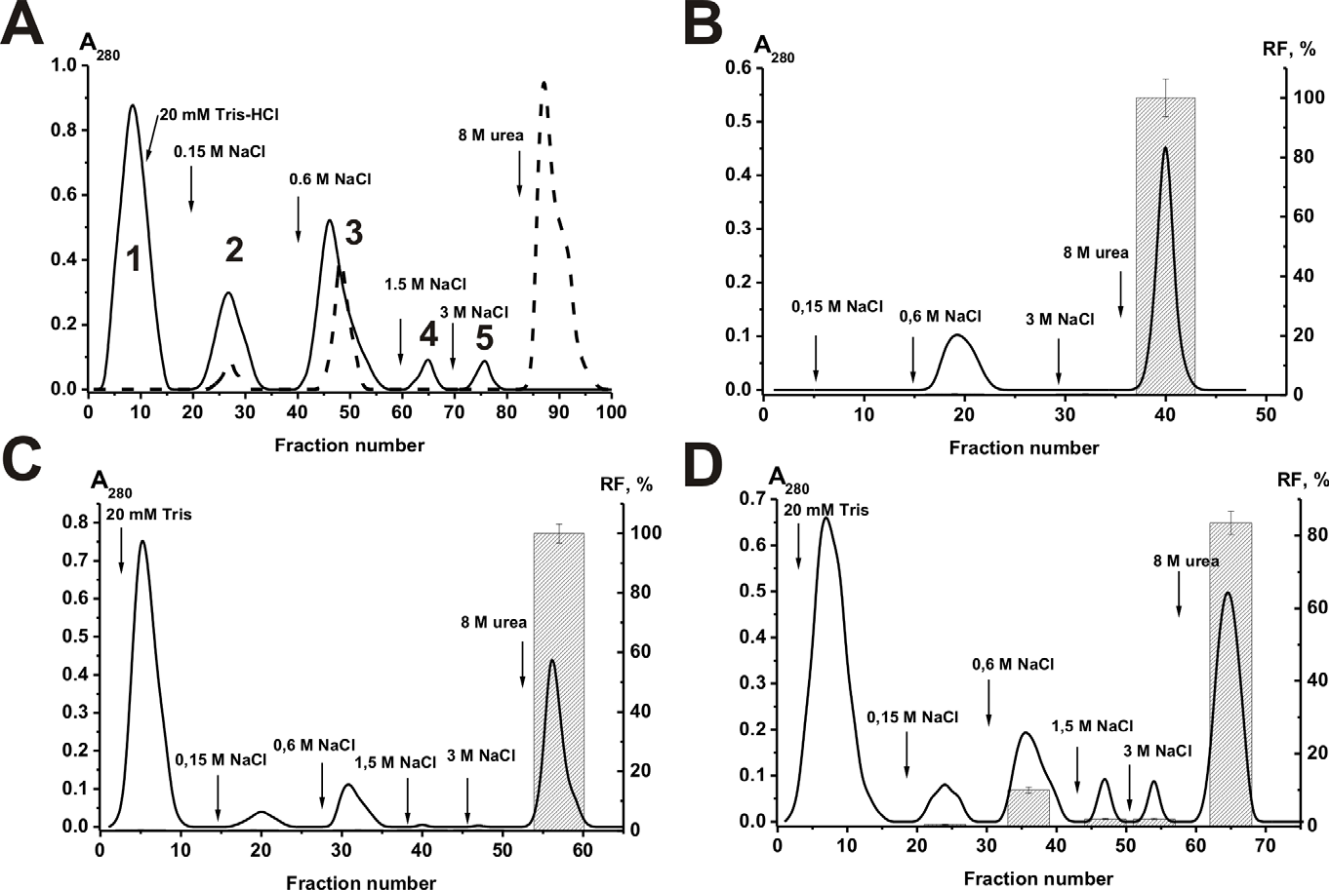
Affinity chromatography of non-modified and FITC-modified sIgA_mix_ on DNA-cellulose: (–) and (- - -), absorbance of sIgA_mix_ at 280 nm before and after modification of sIgAs with FITC, respectively. Analysis of a relative efficiency of specific-molecule exchange under different conditions between non-modified sIgA_mix_ and FITC-sIgA_mix_ having different affinity for DNA-cellulose; relative fluorescence (RF) is shown by the bars (B–D). Before chromatography, the sIgA_mix_ eluted from DNA-cellulose by 0.6 M NaCl (0.6M-IgG_mix_, Panel A) were incubated for 24 h with FITC-IgA_mix_ eluted by 8 M urea (8M-FITC-IgA_mix_, Panel A) in the presence of TBS and GSH (B), TBS + human milk plasma (C), and with this buffer containing both GSH and human milk plasma (D). See Materials and methods for other details.

To analyze an “average” situation of a possible exchange sIgA_mix_ preparation was used. We have separated the sIgA_mix_ before its modification by FITC to five Ab subfractions eluted from DNA cellulose by Tris-buffered saline (TBS; peak 1) or by 0.15–3.0 M NaCl (peaks 2–5) (Fig. 8A). After sIgA_mix_ modification by FITC its affinity for DNA cellulose was increased and only two considerable peaks of Abs correspond to different NaCl concentrations (0.15 and 0.6 M), while the main part of FITC-sIgAs was eluted with 8 M urea. The incubation of non-modified sIgA_mix_ eluted from DNA-cellulose by 0.6 M NaCl (Fig. 8A) with FITC-sIgAs eluted 8 M urea in the buffer containing only reduced glutathione (Fig. 8B) or only milk plasma (Fig. 8C) did not lead to an exchange. The situation was changed dramatically after the addition of both reduced glutathione and milk plasma to the exchange mixtures. As a result of the exchange, after incubation of 0.6M-sIgA_mix_ and 8M-urea-FITC-sIgA_mix_ in the presence of plasma and GSH the FITC-label was distributed between four peaks: all together 14±3% the total FITC-label (average from three experiments) was moved to sIgA_mix_ peaks eluted with 0.6, 1.5, and 3 M NaCl (Fig. 8D). Similar results were obtained in the case of the exchange between non-modified 0.15M-sIgA_mix_ and FITC-labeled 0.6M-sIgA_mix_; 16±4% of the fluorescent label was revealed in Ab peak eluted with 0.15 M NaCl. Thus, after the exchange, approximately 11–20% of FITC-sIgA_mix_ changed the affinity for DNA-cellulose due to formation of less modified sIgAs than that for FITC-labeled 0.6M-sIgA_mix_ and 8M-urea-FITC-sIgA_mix_.

## Discussion

The ability of some Ab molecules to bind a large panel of structurally diverse antigens is known as binding polyspecificity or polyreactivity of Abs. It is common to believe that the antigen-binding ‘pocket’ of many Ab molecules may be flexible and can change conformation to accommodate different antigens which leads to Ab binding polyreactivity. However, affinity of polyreactive Abs for the specific antigens is usually several orders of magnitude higher than their affinity for the non-specific antigens [31], [33]. Some canonical enzymes can also interact with nonspecific ligands [32]. However, the affinity of such enzymes for their specific substrates is usually at least 1–3 orders of magnitude higher than for the nonspecific ligands [32], [35]. It is widely believed that all enzyme-dependent changes in the substrate conformation are necessary for a very precise alignment of electron orbitals of the reacting atoms; it can be achieved only for specific substrates [32], [35]. Therefore, for many enzymes, the conformational adjustment step of the reaction, in contrast to less specific binding, is extremely sensitive to specific elements of the substrate, and it is the catalytic step that determines the reaction rates for different substrates [32], [35]. In contrast to binding, the *k*_cat_ increases by 5–8 orders of magnitude upon a transition from nonspecific ligands to specific substrates [32], [35]. Overall, non-specific binding occurs ubiquitously, while non-specific catalysis is extremely rare.

It was reasonable to expect that all Abs and abzymes nonspecifically interacting with different affinity sorbents can be eluted from the sorbents by ≤0.1–0.5 M NaCl [5], [6], [33]. Taking into account these data and the very high specificity of enzymes at the stage of the catalysis [5], [6], [33], [35], it was surprising that human milk sIgA abzymes catalyzing several very different chemical reactions possessed very high affinity for all affinity sorbents used (Figs. 2, 3, 4, 5). Some fractions of these abzymes could be eluted from all sorbents only in the conditions that destroy exclusively very specific immune complexes. These data could not be explained within the present framework of understanding of Ab binding polyspecificity. Since we have shown that sIgA preparations do not contain any canonical enzymes, the presence of polyfunctional sIgA molecules with different types of HL-fragments in human milk can in principle explain the results of the separation of milk sIgAs on different affinity sorbents (Figs. 2, 3, 4, 5).

The exchange was first demonstrated only between two molecules of IgG4, but not between IgGs of other subclasses [25]–[28]. It was proposed that IgG4 molecules can exchange by half-HL-fragments of the antibodies [25]–[28]. We have recently shown that human milk IgGs to different antigens undergo extensive half-molecule exchange [29]. In the IgGs pool, only 33±5% and 13±5% of Abs contained light chains exclusively of kappa- or lambda-type, respectively, while 54±10% of the IgGs contained both kappa- and lambda-light chains. It addition, it was shown that IgG molecules cannot exchange by only light or heavy chains, which can lead to the formation of Abs with abnormal afunctional combination of variable parts of H- and L-chains to different antigens [29].

A direct way to distinguish sIgA molecules containing two or more different HL-fragments was to reveal a possible existence of chimeric κ-λ-sIgAs. It was shown that only 35±5% and 48±7% of the total sIgAs demonstrated a non-overlapping affinity for light chains of λ- or κ-type, respectively, while 17±4% of the sIgAs effectively interacted with both anti-λ-L-Sepharose and anti-κ-L-Sepharose. Therefore, it was reasonable to suggest that similarly to IgGs [29] an existence of polyfunctional sIgAs is possible in human milk as a result of a some type of specific exchange between λ-λ-, κ-κ-Abs molecules. The question was where and how may occur the formation of λ-κ-chimeric sIgA molecules?

Today the source of IgG in milk is still debated; it may be partially synthesized locally by specific cells of the mammary gland and partially transferred from the mother’s blood circulation system [1], [2]. *In vitro* the extensive exchange of milk IgGs, 25–60%, was found only in the presence of reduced glutathione together with human plasma and it was in agreement with a relative content of chimeric IgGs in fresh milk [29]. It means that specific half-molecule exchange of IgGs can occur directly in the human milk. However, *in vitro* half-molecule exchange of milk sIgAs in the presence of reduced glutathione and human plasma is approximately 1.5–3-fold less intensive than that for IgGs (for example, Fig. 8D). One can suppose that, similarly to IgGs, sIgA molecules in the milk undergo only half-molecule exchange by HL-fragments, but cannot exchange only light or heavy chains. In this connection, it should be mentioned that our sIgA preparations are mixtures of oligomeric ∼370 kDa sIgA1 and ∼300 kDa sIgA2; in sIgA2, the light chains are not linked covalently to the oligomer by disulfide bonds and the light subunits are lost under the harsh conditions of Ab purification (Fig. 1A, lines 1 and 2). Interestingly, we observed no significant exchange between different molecules of the total sIgA preparations containing both sIgA1 and sIgA2 in the absence nor in the presence of GSH only or milk plasma only. Since sIgA2 molecules do not contain light chains, it is likely that *in vitro* only sIgA1 can undergo half-molecule exchange in the presence of GSH and specific enzymes of milk plasma (see below). This is one possible reason behind the lowered exchange efficiency in the case of sIgA in comparison with IgG. However, it does not exclude a possibility of a half-molecule exchange between intact sIgA2 molecules containing light chains in human milk.

It should be mentioned, that we cannot exclude that H_2_L_2_-H_2_L_2_-SJ oligomeric molecules of sIgA1 and sIgA2 can, in principle, go in organisms of mothers through additional exchange by H_2_L_2_-fragments. According to all data obtained including relative activity of sIgAs in catalysis of different reactions after chromatographies on various affinity sorbents, a relative content of milk chimeric sIgAs containing HL-fragments to different antigens may be comparable with that for IgGs. In this connection some other data should be taken into account.

**Figure 9:**
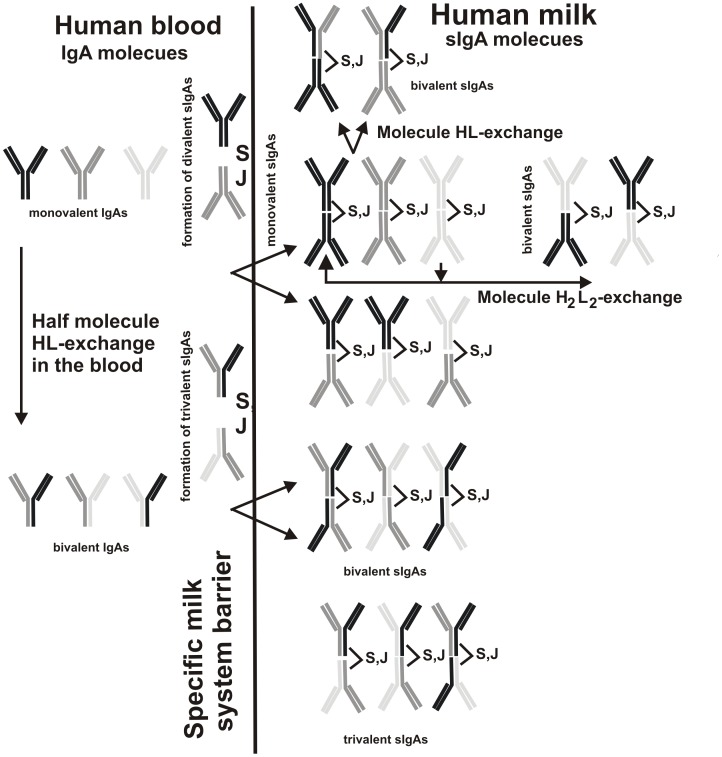
Scheme of a possible (hypothetical) way of formation of chimeric IgA and sIgA as exemplified by three types of H_2_L_2_ IgAs. A half-molecule exchange of IgAs is possible in the blood, while a formation of chimeric di-, tri- and four-valent tetrameric H_4_L_4_SJ molecules of sIgAs from mono- and bivalent IgAs against different antigens may occur under penetration of IgAs through the specific milk barrier, which associates with combination of two Ab molecules with secretory component (S) and join chain (J). In the case of IgAs against three different antigens there may be formation of only mono-, di- and trivalent antibodies, while for four and more different IgAs the formation of tetravalent Abs can occur.

Similarly to IgGs immunoglobulins of A type (IgAs) exist in the human blood as H_2_L_2_ dimers. IgAs are produced by B-lymphocytes of the local immune system of the mammary gland and are present in Payer’s patch lymphoid cells (duodenum), which migrate to mucosal sites, where on the specific milk barrier they associate with secretory component (S) and join chain (J) forming tetrameric H_4_L_4_SJ sIgA oligomers. We have shown recently that the pool of blood sera IgGs of healthy donors contains approximately from 4 to 8% of chimeric λ-κ-IgGs (Sedykh and Nevinsky; personal communication). Therefore, we can suppose that similarly to IgGs small percent of IgAs may undergo a half-molecule exchange even in the blood before migration to mucosal system; Fig. 9 schematically shows such a type of a possible (hypothetical) exchange. In addition, one cannot exclude that during penetration through the specific milk barrier secretory component can combined molecules of dimeric IgAs against very different antigens as well as IgAs containing light chains of lambda and kappa types (Fig. 9). Thus, we can suppose that human milk can contain oligomeric sIgA molecules containing HL fragments from one up to four different antigens (Fig. 9). In this case due to high affinity of one HL fragment to one of many different antigens (DNA, ATP, oligosaccharide, lipid, protein et al.) some sIgA molecules can interact with affinity sorbents bearing one immobilized antigen, while from one to three of other HL fragments can efficiently interact with other different antigens including following catalysis in the case of HL fragments possessing catalytic activities. This assumption is consistent with data on the chromatography and re-chromatography of sIgAs on various affinity sorbents and distribution of all activities across the profile of every of these chromatographies (Figs 2, 3, 4, 5) as well as with the existence of chimeric λ-κ-sIgAs.

Since the extensive exchange occurs only in the presence of GSH together with milk plasma, it is reasonable to suggest that some milk components, most probably protein(s) and/or enzyme(s) (similarly to disulphide isomerase and/or FcRn in the case of IgG4 [25]–[28]) can stimulate the exchange.

The phenomenon of the exchange should lead to an increase in polyspecificity of polyclonal Abs and to cross-catalytic activity observed by us for the first time for milk IgGs and sIgAs.

## Materials and Methods

### Chemicals and Donors

Most of the reagents including monoclonal mouse Abs (anti-kappa-Abs and anti-lambda-Abs) used in this work were obtained from Sigma. SDS was obtained from Merck. We also used Protein G-Sepharose and Protein A-Sepharose from GE Healthcare. Glutaraldehyde-crosslinked DNA-cellulose was from NIKTI BAV (Russia). Sepharoses bearing the various monoclonal Abs, β-casein, or γ-(aminohexamethylenamide)-ATP (ATP-Sepharose) were prepared using BrCN-activated Sepharose according to the standard manufacturer’s protocol. Samples of milk were taken by obstetricians from five healthy mothers (19–35-years old) within the period of 1*–*3 weeks after the beginning of lactation during standing in maternity hospital. The milk sampling protocol conformed to the local human ethics committee guidelines (Ethics committee of Novosibirsk State Medical University, Novosibirsk, Russia) including written consent of women recently confined to present of an excess of their milk for scientific purposes. According to standard procedure in Russian hospitals all pregnant women before the admission to the maternity hospital should be analyzed for different possible diseases in accordance with Helsinki ethics committee guidelines. Obstetrician-gynecologists gave us milk samples from donors having a negative history of autoimmune, rheumatologic, respiratory, cardiovascular, gastrointestinal, reproductive, or nervous system pathology.

### Purification and Analysis of Antibodies

Five electrophoretically and immunologically homogeneous sIgA preparations were obtained by affinity chromatography of breast milk protein on Protein A-Sepharose (after removing of IgGs using Protein G-Sepharose as in [14]–[16]) followed by gel filtration on a Superdex 200 HR 10/30 column under the conditions that remove non-specifically bound proteins similarly to [18]–[20]. The SDS-PAGE analysis of the Ab fractions for homogeneity under nonreducing conditions was performed in a 4–16% gradient gel (0.1% SDS); an analysis for the polypeptide spectrum was performed in a reducing 12.5% gel (in the presence of 0.1% SDS and 10 mM DTT) in the Laemmli system as described previously [16]–[23]. The gel was silver-stained according to a standard procedure. The type of Abs (sIgA, IgG or IgM) in the fractions was determined by Western blotting on a nitrocellulose membrane as described previously [18]. In order to protect the sIgA preparations from bacterial contamination, they were filtered through a Millex filter (pore size 0.2 µm). After 1 week of storage at 4°C for refolding after the “acidic shock”, a necessary step in the purification, the sIgAs were used in the activity assays as described below. To exclude possible artifacts due to traces of contaminating enzymes, the sIgA activities in the hydrolysis of DNA, ATP, oligosaccharides, phosphorylation of casein, lipids and oligosaccharides were analyzed after SDS-PAGE of sIgAs as in [16]–[23] (see below).

### DNase Activity Assay

DNase activity was analyzed using supercoiled pBluescript plasmid DNA as described earlier [14]–[16]. The reaction mixture (20 µl) contained 50 mM Tris-HCl (pH 7.5), 5 mM MgCl_2_, 20 µg/ml DNA, 1 mM EDTA, and 5–30 µg/ml sIgAs, and was incubated for 0.5–3 h (standard time, 2 h) at 37°C. The cleavage products were analyzed by electrophoresis in a 1% agarose gel. The images of ethidium bromide-stained gels were captured on a Sony DSC-F717 camera and a relative amount of DNA in different bands was analyzed using ImageQuant v5.2 (Molecular Dynamics). The activities of sIgAs were determined as a decrease in the percentage of DNA converted from the initial supercoiled form to the relaxed form (and sometimes additionally linear form), corrected for the distribution of DNA between these bands in the control (incubation of pBluescript in the absence of Abs). All measurements (initial rates) were taken within the linear regions of the time courses and Ab concentration curves.

### ATP-hydrolyzing Activity Assay

ATPase activity was analyzed as in [18]. The reaction mixtures (20 µl) containing 50 mM Tris-HCl (pH 7.5), 1 mM MgCl_2_, 0.3 mM EDTA, 0.2 mM γ-[^32^P]ATP (1×10^7^ cpm) and 20–80 µg/ml sIgAs were incubated for 1–3 h at 37°C (standard time, 2 h). The products of ATP hydrolysis were analyzed by thin-layer chromatography on PEI-cellulose plates (Merck) in 0.25 M potassium phosphate (pH 7.0). After the chromatography, the plates were dried and various ^32^P-labeled products were quantified by phosphorimaging (Molecular Imager FX system, Bio-Rad Laboratories, Hercules, CA). The relative amounts of radioactivity in the products were calculated using the scanning data of the spots corresponding to [^32^P]*ortho*phosphate and non-hydrolyzed γ-[^32^P]ATP. The activities of sIgAs were determined from percentages of the product in the spots of [^32^P]*ortho*phosphate and non-hydrolyzed γ-[^32^P]ATP. All measurements were taken within the linear regions of the time courses and Ab concentration dependences.

### Amylase Activity Assay

Amylolytic activity was analysed as in [17]. The reaction mixture (15–20 µl) containing 30 mM Tris-HCl (pH 7.5), 1 mM NaN_3_, 1.5 mM maltoheptaose, and 20–80 µg/ml of sIgAs was incubated for 6–12 h at 30°C. Products of hydrolysis were identified by TLC on Kieselgel plates (Merck) using 1-butanol–acetic acid–H_2_O (12∶4∶4). The activities of sIgAs were determined from the scanning data from percentages of oligosaccharides in the spots of maltoheptaose and its hydrolyzed forms. All measurements were taken within the linear regions of the time courses and Ab concentration dependences.

### Protein Kinase Activity Assay

Protein kinase activity of milk sIgAs was measured as in [20]. The reaction mixtures (20 µl) contained 50 mM Tris-HCl (pH 6.8), 3 mM MgCl_2_, 0.3 mM EDTA, 50 mM NaCl, 0.6 mg/ml casein, 0.1 mM γ[^32^P]ATP (5×10^7^ cpm), and 50–200 µg/ml sIgAs. The product of casein phosphorylation was analyzed by Laemmli SDS-PAGE in a 12.5% gel with Coomassie R250 staining. The relative amount of [^32^P]casein was quantified by phosphorimaging.

### Assay of Lipid and Oligosaccharide Kinase Activities of sIgAs

Phosphorylation of lipids and oligosaccharides tightly bound to the sIgAs was analyzed as in [21]–[23]. The reaction mixtures (20 µl) contained 10 mM Tris-HCl (pH 7.5), 1 mM MgCl_2_, 0.1 mM EDTA, 70 mM NaCl, 0.1 µM [^32^P]*ortho*phosphate (20 µCi), and 20–100 µg/ml sIgAs. The samples were incubated at 37°C for 2 h. The reaction was stopped by addition of an equal volume of 20% trichloroacetic acid (20 µl), and [^32^P]lipids was extracted with a chloroform–methanol mixture (2∶1). The extracts were evaporated to dryness, the lipids were solved in 10 µl of a chloroform–methanol mixture (1∶1) and analyzed by TLC on Kieselgel 60 plates using the solvent system A, chloroform–methanol–H_2_O (14∶6∶1) [21]–[23]. The aqueous phase of the extracted solution containing [^32^P]oligosaccharides was dried, the precipitate was solved in 5–10 µl of water and used for the analysis of [^32^P]oligosaccharides by TLC on Kieselgel 60 plates using the solvent system B, dioxane–7 M NH_4_OH–H_2_O (5∶1∶4) [21]–[23]. The relative amounts of [^32^P]lipids and [^32^P]oligosaccharides were quantified by phosphorimaging.

### Chromatography of sIgAs on DNA-cellulose

Electrophoretically homogeneous sIgAs were loaded onto a DNA cellulose column (14 ml) equilibrated with 20 mM Tris-HCl (pH 7.5), and the column was washed with the same buffer to zero optical density. The sIgAs were eluted using the same buffer and either a gradient of NaCl concentration (0–3 M) or different concentrations of NaCl (0.02, 0.15, 0.3, 1.5, 3.0 M), and then with 3 M MgCl_2_, as in [14], [15]. sIgA fractions were collected, dialyzed against 20 mM Tris-HCl (pH 7.5) containing 0.1 M NaCl, concentrated, and each fraction was used in the analysis of various enzymatic activities.

### Chromatography of sIgAs on ATP-Sepharose

Purified sIgAs were applied to an ATP-Sepharose column (3 ml) equilibrated with 25 mM Tris-HCl (pH 7.5) containing 1 mM MgCl_2_ as in [20]. Unbound proteins were eluted with the same buffer. Adsorbed sIgAs were eluted with a gradient of NaCl concentration (0–3 M) in 25 mM Tris-HCl (pH 7.5), and then with 3 M MgCl_2_. Individual fractions were collected, dialyzed against 20 mM Tris-HCl (pH 7.5), concentrated, and their catalytic activities were measured as described above.

### Chromatography of sIgAs on Casein-Sepharose

Purified sIgAs was subjected to a chromatography on a casein-Sepharose column (7 ml) equilibrated in 20 mM Tris-HCl (pH 7.5) similarly to [19]. After sIgA loading, the column was washed with this buffer to zero optical density in the eluate. The bound sIgAs were eluted with with the same buffer containing different concentrations of NaCl (50, 150, and 3 M), and then with 3 M MgCl_2_. sIgAs were collected, dialyzed against 10 mM Tris-HCl (pH 7.5) containing 0.1 M NaCl, concentrated, and each fraction was used in the assay of various catalytic activities.

### Chromatography of sIgAs on Lipid-saturated Silicagel

The sorbent was prepared by saturation of silicagel with a chloroform-methanol (1∶1) extract of the human milk lipid and fat fraction. Purified sIgAs were then applied to the column (10 ml) equilibrated in 20 mM Tris-HCl (pH 7.5) as in [29]. Unbound proteins were eluted with the same buffer. The bound sIgAs were eluted with with the same buffer containing different concentrations of NaCl (0.05, 0.15, and 1.2 M). sIgA fractions were collected, dialyzed against 10 mM Tris-HCl (pH 7.5), concentrated, and each fraction was used in the assay of various catalytic activities.

### SDS-PAGE Assay of DNase Activity

The DNase activity of sIgA after SDS-PAGE was analyzed in a gel containing calf thymus DNA (5 µg/ml) under non-reducing conditions as in [14]–[16]. Before the electrophoresis, the sIgA_mix_ samples were incubated at 22°C for 10–20 min in 20 mM Tris-HCl (pH 7.5) containing 0.1% SDS. To restore the enzymatic activity after SDS-PAGE, SDS was removed by incubating the gel for 1 h at 22°C in 20 mM Tris-HCl (pH 7.5) and washing the gel five times with the same buffer. To refold the protein after SDS treatment and to assay for DNase activity, longitudinal slices of the gel were incubated at 25°C for 15–48 h in the reaction buffer containing 20 mM Tris-HCl (pH 7.5), 4 mM MgCl_2_, and 0.2 mM CaCl_2_. To visualize the products of DNA hydrolysis, the gel was stained with ethidium bromide. The same ethidium bromide-stained or parallel longitudinal slices were used to detect the position of sIgA in the gel by Coomassie Blue staining.

### SDS-PAGE Analyses of ATPase and Amylolytic Activities

For SDS-PAGE assay of ATPase and amylase activities, sIgAs (7–10 µg) were preincubated at 30°C for 30 min under nonreducing conditions (50 mM Tris-HCl, pH 7.5, 1% SDS, and 10% glycerol) as in [17]–[18]. After the electrophoresis, SDS was removed by incubating the gel for 30 min at 30°C with H_2_O (5 times). To restore the enzymatic activity after SDS-PAGE, SDS was removed by incubating the gel for 1 h at 22°C with K-phosphate (pH 6.8). The gel was washed five times with this buffer. Then 3–4-mm cross-sections of longitudinal slices of the gel were cut out and incubated with 50 µl of 20 mM Tris-HCl, pH 7.5, containing 5 mM MgCl_2_ and 1 mM EDTA for two days at 4°C to allow protein refolding and eluting from the gel. The solutions were removed from the gels by centrifugation and used for assay of ATP and oligosaccharide hydrolysis as described above. Parallel longitudinal lanes were used for detecting the position of sIgA in the gel by Coomassie R250 staining.

### Purification of Lambda-sIgAs, Kappa-sIgAs, and Lambda-kappa-sIgAs

sIgAs (0.1–1.0 mg) were chromatographed on Sepharose bearing immobilized monoclonal mouse specific Abs to human kappa- or lambda-light chains of Abs. The column (1 ml) was equilibrated with 50 mM Tris-HCl (pH 7.5) containing 50 mM NaCl; the protein was applied and then the column was washed with a buffer containing 0.5 M NaCl to zero optical density. sIgAs were eluted from the sorbent with 0.1 M glycine-HCl (pH 2.6). The column fractions were collected into cooled tubes containing 50 µl of 0.5 M Tris-HCl (pH 9.0), and were additionally neutralized with this buffer. The fractions having affinity for anti-kappa-Abs were re-chromatographed on anti-λ-L-Sepharose, while sIgAs eluted from anti-λ-L-Sepharose, on anti-κ-L-Sepharose. The final fractions were dialyzed against 50 mM Tris-HCl (pH 7.5) containing 50 mM NaCl and concentrated. In order to protect Ab preparations from bacterial contamination, all fractions used were filtered through a Millex syringe-driven filter units (0.2 µm) and kept in sterilized tubes. The preparations obtained were used for ELISA and determination of their catalytic activities.

### Analysis of Effect of sIgA Purification Conditions on the Exchange Reaction

Re-purification of the mixture of lambda- and kappa-sIgAs was performed similarly to purification of sIgAs from human milk (see above). A mixture (1 ml) of equal amounts of purified λ- and κ-sIgAs without admixture of chimeric λ-κ-sIgAs containing 150 mM NaCl, 50 mM Tris-HCl, pH 7.5 and 2.2 mg/ml λ+κ-sIgA_mix_ was incubated at 25°C for 24 h. Then, it was diluted 5 times with buffer A (150 mM NaCl, 50 mM Tris-HCl, pH 7.5) and loaded on a 3-ml protein G-Sepharose column equilibrated in buffer A. The flow-through fraction was loaded on a 3-ml protein A-Sepharose column equilibrated in buffer A. The column was washed with 8 ml of buffer A and then with this buffer (5 ml) containing 1% Triton X-100 and 0.3 M NaCl and the column was washed with buffer A to zero optical density. The total sIgA_mix_ fraction was eluted in 0.1 M glycine-HCl (pH 2.6), the column fractions were collected to cooled tubes containing 50 µl of 0.5 M Tris-HCl (pH 9.0) and concentrated for the following step of purification. FPLC gel filtration of this fraction was performed on a Superdex 200 HR 10/30 column as in [20]–[22]. The column fractions were collected, neutralized, and dialyzed as described above for sIgA purification from human milk; sIgA_mix_ was used for an analysis of the content of λ-, κ-, and λ-κ-sIgAs.

### ELISA of Different Antibodies

After chromatography of sIgAs on Sepharose bearing immobilized monoclonal mouse anti-kappa- or anti-lambda-Abs, the sIgAs were analyzed for the content of κ-sIgAs, λ-sIgAs, and λ-κ-sIgAs by ELISA. For ELISA, sodium carbonate (50 µl, pH 9.6) containing 0.4–12 µg/ml of one of the tested sIgAs was added to the ELISA strips and incubated overnight at 22°C. The assembled strips were washed once with TBS buffer containing 0.01% NaN_3_ and 0.05% Triton X-100 and then twice with the same buffer without Triton X-100. The strips were blocked for 2 h at 37°C using TBS containing 3% bovine albumin and 0.01% NaN_3_, and washed 10 times with water and then with TBS containing 0.01% NaN_3_.

Each of the monoclonal mouse Abs (100 µl, 0.01 mg/ml; anti-κ-IgG, or anti-λ-IgG) in TBS containing 3.0% bovine albumin, 0.01% NaN_3_ and 0.05% Triton X-100 was added to the strips corresponding to human sIgAs of different types and incubated for 2 h at 37°C. After washing the strips with water (10 times) and TBS, 100 µl TBS containing 1.0% bovine albumin and 0.01% NaN_3_ were added and incubated for 2 h at 37°C. The strips were washed 10 times with water, incubated with 100 µl TBS containing 1 µg/ml conjugate of polyclonal rabbit anti-mouse IgGs with horseradish peroxidase for 30 min at 37°C, and washed again 10 times with water. After an addition of 50 µl of citrate/phosphate buffer containing 3,3',5,5'-tetramethylbenzidine and H_2_O_2_, the strips were incubated for 15 min at room temperature, and the reaction was stopped by addition of 100 µl of 1 M H_2_SO_4_. The relative concentration of analyzed Abs in the samples was expressed as the difference in the relative absorbance at 450 nm (average of three measurements) between the experimental and control data.

### Preparation of Milk Plasma

Milk (10 ml) from healthy mothers was centrifuged for 1 h at 6000×*g* as in [29]. The lipid and cell phases were removed, the solution was dialyzed against TBS (150 mM NaCl, 20 mM Tris-HCl pH 7.4), and then filtered twice through Sephadex G-75 (10 ml) to remove fats that have remained in the solution. Then, all Abs were removed from the milk plasma using a sequential affinity chromatography of plasma proteins on protein G-Sepharose (5 ml) and protein A-Sepharose (5 ml) equilibrated with TBS. The flow-through fraction from the affinity sorbents data did not contain Abs according to ELISA.

### Preparation of Labeled sIgAs

To obtain sIgA_mix_ preparation modified with fluorescein isothiocyanate (FITC), the reaction mixture (1 ml) containing 0.1 M NaHCO_3_ (pH 8.3), 0.1 mg/ml FITC, and 1–2 mg/ml sIgA_mix_ was incubated for 36 h at 24°C in darkness. FITC-sIgAs were purified by gel filtration on Sephadex G-25 Superfine (1×10 cm) equilibrated with 30 mM Tris-HCl (pH 7.5). Then the FITC-sIgA preparation was separated for several fractions with different affinity for DNA by chromatography on DNA-cellulose similarly to non-modified sIgAs (see above). FITC-sIgA fraction eluted with NaCl and 8 M urea was dialyzed using 50 mM Tris-HCl buffer, pH 7.5. The fluorescence of FITC-sIgAs was analyzed using a PharosFX imaging system (Bio-Rad; fluorophores mode, FITC, high sample intensity). After concentration, the FITC-sIgA preparations were used for the analysis of sIgA exchange.

### sIgA Exchange Analysis

The reaction mixtures (0.2–2.0 ml) contained 20 mM Tris-HCl (pH 7.5), 0.15 M NaCl, 10–100 mM GSH, equal amounts of labeled and non-labeled sIgAs (0.5–2 mg/ml) possessing different affinity for DNA-cellulose, and human plasma containing no Abs (1/10 of the total volume). The mixtures were incubated for 48 h at 37°C in darkness and then dialyzed against 20 mM Tris-HCl. To reduce the disulfide bonds of sIgAs, oxidized glutathione was added to 100 mM final concentration, and the reaction mixture was incubated for 24 h at 37°C. Then the reaction mixtures were loaded onto a DNA-cellulose column (7 ml) equilibrated with 20 mM Tris-HCl (pH 7.5), and the column was washed with the same buffer to zero optical density. The sIgAs were eluted using the same buffer containing NaCl (0.15, 0.3, 1.5, and 3.0 M). sIgA fractions were collected, dialyzed against 20 mM Tris-HCl (pH 7.5), and their relative fluorescence was determined.

## References

[1] Fey HR, Burtler R, Marti F (1973) The production in the pregnant cow of anti-human immunoglobulin to be used for the antiglobulin test. Vox Sang 25: 245–253. 4200729 10.1111/j.1423-0410.1973.tb04369.x

[2] Mestecky J, McGhee JR (1987) Immunoglobulin A (IgA): molecular and cellular interactions involved in IgA biosynthesis and immune response. Adv. Immunol. 40: 153–245. 10.1016/s0065-2776(08)60240-0 3296685

[3] Hanson LA, Hahn-Zoric M, Berndes M, Ashraf R, Herias V, et al (1994) Breast feeding: overview and breast milk immunology. Acta Paediatr Jpn 36: 557–561. 7825463 10.1111/j.1442-200x.1994.tb03246.x

[4] Keinan E ed. (2005) Catalytic antibodies: Weinheim, Germany: Wiley-VCH Verlag GmbH and Co. KgaA. 1–586 p.

[5] Nevinsky GA, Buneva VN (2005) Natural catalytic antibodies - abzymes. In: Keinan E editor. Catalytic antibodies. Weinheim, Germany: VCH-Wiley press. 503–567.

[6] Nevinsky GA (2010) Natural catalytic antibodies in norm and in autoimmune diseases. In: ed Brenner KJ editor. Autoimmune Diseases: Symptoms, Diagnosis and Treatment. USA: Nova Science Publishers, Inc. 1–107.

[7] Amino N, Mori H, Iwatani Y, Tanizawa O, Kawashima M, et al (1982) High prevalence of transient post-partum thyrotoxicosis and hypothyroidism. N Engl J Med 306: 849–852. 7062963 10.1056/NEJM198204083061405

[8] Tanaka A, Lindir R, Gershwin VE (2000) Fetal microchimerisms in the mother: immunologic implications. Liver Transpl 6: 138–143. 10719011 10.1002/lt.500060225

[9] Nevinsky GA (2011) Natural catalytic antibodies in norm and in HIV-infected patients. In: Fyson Hanania Kasenga editor. Understanding HIV/AIDS Management and Care – Pandemic Approaches the 21st Century. InTech. 151–192.

[10] Paul S, Volle DJ, Beach CM, Johnson DR, Powell MJ, et al (1989) Catalytic hydrolysis of vasoactive intestinal peptide by human autoantibody. Science 244: 1158–1162. 2727702 10.1126/science.2727702

[11] Kalaga R, Li L, O’Dell JR, Paul S (1995) Unexpected presence of polyreactive catalytic antibodies in IgG from unimmunized donors and decreased levels in rheumatoid arthritis. J Immunol 155: 2695–2702. 7650397

[12] Izadyar L, Friboulet A, Remy MH, Roseto A, Thomas D (1993) Monoclonal anti-idiotypic antibodies as functional internal images of enzyme active sites: production of a catalytic antibody with a cholinesterase activity. Proc Natl Acad Sci USA 90: 8876–8880. 8415624 10.1073/pnas.90.19.8876 PMC47463

[13] Kolesnikov AV, Kozyr AV, Alexandrova ES, Koralewski FK, Demin AV, et al (2000) Enzyme mimicry by the antiidiotypic antibody approach. Proc Natl Acad Sci USA 97: 13526–13531. 11095704 10.1073/pnas.200360497 PMC17609

[14] Kanyshkova TG, Semenov DV, Khlimankov D, Buneva VN, Nevinsky GA (1997) DNA-hydrolyzing activity of the light chain of IgG antibodies from milk of healthy human mothers. FEBS Lett 416: 23–26. 9369225 10.1016/s0014-5793(97)01163-0

[15] Buneva VN, Kanyshkova TG, Vlassov AV, Semenov DV, Khlimankov DY, et al (1998) Catalytic DNA- and RNA-hydrolyzing antibodies from milk of healthy human mothers. Appl Biochem Biotechnol 75: 63–76. 10214697 10.1007/BF02787709

[16] Nevinsky GA, Kanyshkova TG, Semenov DV, Vlassov AV, Gal’vita, et al (2000) Secretory immunoglobulin A from healthy human mothers’ milk catalyzes nucleic acid hydrolysis. Appl Biochem Biotechnol 83: 115–129. 10826954 10.1385/abab:83:1-3:115

[17] Savel’ev AN, Kanyshkova TG, Kulminskaya AA, Buneva VN, Eneyskaya EV, et al (2001) Amylolytic activity of IgG and sIgA immunoglobulins from human milk. Clin Chim Acta 314: 141–152. 11718689 10.1016/s0009-8981(01)00691-x

[18] Semenov DV, Kanyshkova TG, Karotaeva NA, Krasnorutskii MA, Kuznetsova IA, et al (2004) Catalytic nucleotide-hydrolyzing antibodies in milk and serum of clinically healthy human mothers. Med Sci Monit 10: BR23–BR33. 14737037

[19] Odintsova ES, Zaksas NP, Buneva VN, Nevinsky GA (2011) Metal dependent hydrolysis of β-casein by sIgA antibodies from human milk. J Mol Recognit 24: 45–59. 20140974 10.1002/jmr.1022

[20] Nevinsky GA, Kit YY, Semenov DV, Khlimankov DY, Buneva VN (1998) Secretory immunoglobulin A from human milk catalyzes milk protein phosphorylation. Appl Biochem Biotechnol 75: 77–91. 10214698 10.1007/BF02787710

[21] Gorbunov DV, Karataeva NA, Buneva VN, Nevinsky GA (2005) Lipid kinase activity of antibodies from milk of clinically healthy human mothers. Biochim Biophys Acta 1735: 153–166. 16039903 10.1016/j.bbalip.2005.06.007

[22] Karataeva NA, Gorbunov D, Prokudin IV, Buneva VN, Kulminskaya AA, et al (2006) Human milk antibodies with polysaccharide kinase activity. Immunol Lett 103: 58–67. 16313972 10.1016/j.imlet.2005.10.009

[23] Karataeva NA, Buneva VN, Nevinsky GA (2006) Polysaccharide kinase activity of human milk IgG antibodies. Biochemistry (Mosc.) 71: 1207–1221. 17140382 10.1134/s000629790611006x

[24] Buneva VN, Kudryavtseva AN, Gal’vita AV, Dubrovskaya VV, Khokhlova OV, et al (2003) Dynamics of antibody nuclease activity in blood of women during pregnancy and lactation. Biochemistry (Moscow) 68: 890–900. 12948390 10.1023/a:1025703132523

[25] Rob C, Aalberse RC, Schuurman J (2002) IgG4 breaking the rules. Immunology 105: 9–19. 11849310 10.1046/j.0019-2805.2001.01341.x PMC1782638

[26] Van der Neut Kolfschoten M, Schuurman J, Losen M, Bleeker WK, Martínez-Martínez P, et al.. (2007) Anti-inflammatory activity of human IgG4 antibodies by dynamic Fab arm exchange. Science 317, 1554–1557. 10.1126/science.1144603 17872445

[27] Schuurman J, Van Ree R, Perdok GJ, Van Doorn HR, Tan KY, et al (1999) Normal human immunoglobulin G4 is bispecific: it has two different antigen-combining sites. Immunology 97: 693–698. 10457225 10.1046/j.1365-2567.1999.00845.x PMC2326875

[28] Rispens T, den Bleker TH, Aalberse RC (2010) Hybrid IgG4/IgG4 Fc antibodies form upon ‘Fab-arm’ exchange as demonstrated by SDS-PAGE or size-exclusion chromatography. Mol Immunol 47: 1592–1594. 20299101 10.1016/j.molimm.2010.02.021

[29] Sedykh SE, Buneva VN, Nevinsky GA (2012) Human milk IgGs contain various combinations of different antigen-binding sites resulting in multiple variants of their bispecificity, PLOS ONE. 7(8): e42942. 10.1371/journal.pone.0042942 PMC3418227 22912765

[30] Mestecky J, Russell MW, Jackson S, Brown TA (1986) The human IgA system: a reassessment. Clin Immunol Immunopathol 40: 105–114. 2424650 10.1016/0090-1229(86)90073-5

[31] Notkins AL (2004) Polyreactivity of antibody molecules. Trends in Immunology 25: 174–179. 15039043 10.1016/j.it.2004.02.004

[32] Nevinsky GA (2003) In:Uversky VN editor. Protein Structures: Kaleidoscope of Structural Properties and Functions. Kerala: Research Signpost. Pp. 133–222, 2003.

[33] Andryushkova AA, Kuznetsova IA, Orlovskaya IA, Buneva VN, Nevinsky GA (2009) Nucleotide-hydrolyzing antibodies from the sera of autoimmune-prone MRL-lpr/lpr mice. Int Immunol 21: 935–945. 19556305 10.1093/intimm/dxp060

[34] Zhou Z-H, Tzioufas AG, Notkins AL (2007) Properties and function of polyreactive antibodies and polyreactive antigen-binding B cells. J Autoimmun 29: 219–228. 17888628 10.1016/j.jaut.2007.07.015 PMC2100422

[35] Fersht A (1977) Enzyme Structure and mechanism: San Francisco: W.H. Freeman and Co.

